# Zinc in Infection and Inflammation

**DOI:** 10.3390/nu9060624

**Published:** 2017-06-17

**Authors:** Nour Zahi Gammoh, Lothar Rink

**Affiliations:** Institute of Immunology, Faculty of Medicine, RWTH Aachen University, University Hospital, Pauwelstrasse 30, 52074 Aachen, Germany; nour.gammoh@rwth-aachen.de

**Keywords:** zinc, infection, inflammation, homeostasis

## Abstract

Micronutrient homeostasis is a key factor in maintaining a healthy immune system. Zinc is an essential micronutrient that is involved in the regulation of the innate and adaptive immune responses. The main cause of zinc deficiency is malnutrition. Zinc deficiency leads to cell-mediated immune dysfunctions among other manifestations. Consequently, such dysfunctions lead to a worse outcome in the response towards bacterial infection and sepsis. For instance, zinc is an essential component of the pathogen-eliminating signal transduction pathways leading to neutrophil extracellular traps (NET) formation, as well as inducing cell-mediated immunity over humoral immunity by regulating specific factors of differentiation. Additionally, zinc deficiency plays a role in inflammation, mainly elevating inflammatory response as well as damage to host tissue. Zinc is involved in the modulation of the proinflammatory response by targeting Nuclear Factor Kappa B (NF-κB), a transcription factor that is the master regulator of proinflammatory responses. It is also involved in controlling oxidative stress and regulating inflammatory cytokines. Zinc plays an intricate function during an immune response and its homeostasis is critical for sustaining proper immune function. This review will summarize the latest findings concerning the role of this micronutrient during the course of infections and inflammatory response and how the immune system modulates zinc depending on different stimuli.

## 1. Introduction

Zinc is a nutritionally fundamental trace element and is the second most abundant trace metal in the human body after iron. The influence of zinc on human health was first observed and described by Prasad et al. in the 1960s [1]. Zinc research has come a long way since then; its significance as a structural component in many proteins and its participation in numerous cellular functions is now well established. Such functions include, but are not limited to, cell proliferation and differentiation [2], RNA and DNA synthesis [3], as well as cell structures and cell membrane stabilization [4]. Its multifaceted role in the regulation of the immune system is particularly interesting and will be discussed in more detail in this review [5]. Consequently, zinc’s implication in an array of functions demonstrates how a defect in nutritional absorption may lead to the manifestation of various diseases.

Zinc is involved in many metabolic and chronic diseases such as: diabetes, cancer (esophageal, oral small cell carcinoma, breast cancer), and neurodegenerative diseases. There is also strong evidence between zinc deficiency and several infectious diseases such as malaria, HIV, tuberculosis, measles, and pneumonia [6].

## 2. Zinc and Nutrition

The total zinc content in the human body amounts to 2–4 g, with a plasma concentration of 12–16 μM [2]. Albeit it is a small plasma pool, it is rapidly exchangeable and mobile. Sufficient daily intake of zinc is necessary to maintain a steady state because, unlike iron, the body has no specialized zinc storage system. The highest concentrations of zinc are found in the muscles, bones, skin, and liver [7,8]. 

Recommended daily intake of zinc depends on several factors such as age, sex, weight, and phytate content of diet. Those recommended values differ in each country. The US Food and Nutrition Board recommended intake of 11 mg/day and 8 mg/day for adult males and females, respectively [9]. While the German Society of Nutrition’s recommendation comprised of 10 mg/day and 7 mg/day for adult males and females, respectively [10]. Both the World Health Organization (WHO) and the European Food Safety Authority (EFSA) consider the inhibitory effect of dietary phytate on zinc absorption when setting the recommended zinc intake values. WHO categorizes diets according to their potential absorption efficiency of zinc, per phytate-zinc molar ratio, into 3 groups; high (<5), moderate (5–15), and low (>15) zinc bioavailability [11]. Whereas EFSA provides different zinc reference recommendations for diets containing phytate intake levels of 300, 600, 900, and 1200 mg/day [12].

As mentioned, zinc bioavailability depends on the composition of the diet. Non-digestible plant ligands such as phytate, some dietary fibers, and lignin chelate zinc and inhibit its absorption. Other factors that influence the absorption of zinc are calcium and iron. Zinc is present in many food groups and its concentration and bioavailability varies considerably. Foods with the highest zinc concentration include red meat, some shellfish, legumes, fortified cereals, and whole grains [9]. Zinc from animal sources has higher bioavailability compared to zinc sourced from plant products. People who abstain from eating red meats, vegetarians, vegans, and people living in developing country who rely mainly on plant-based foods are at higher risk of developing zinc deficiency due to inadequate zinc intake [13]. Oral zinc supplements are readily available but not all offer the same zinc bioavailability. Zinc bound to amino acids such as aspartate, cysteine, and histidine shows the highest absorption concentration, followed by zinc chloride, sulfate, and acetate, whereas zinc oxide show the lowest bioavailability [14].

According to the WHO, zinc deficiency is currently the fifth leading cause of mortality and morbidity in developing countries. It is estimated that it affects about one-third of the world’s population. Worldwide, zinc deficiency accounts for approximately 16% of lower respiratory tract infections, 18% of malaria, and 10% of diarrheal diseases. While severe zinc deficiency is rare, mild to moderate deficiency is more common worldwide [15,16].

## 3. Zinc Homeostasis

### 3.1. Zinc Transporters

There are two major protein families that mammalian zinc transporters belong to. The first group of transporter are ZIP (Zrt/Irt-like proteins), which are responsible for transporting zinc into the cytosol from either extracellular space or from intracellular compartments. There are 14 ZIP transporters, designated as Solute Carrier family SLC39A1-A14. The second group of 10 transporters are ZnT (Zinc transporters), which are designated as SLC30A1-A10. They generally transport zinc out of the cytosol into extracellular space or intracellular organelles such as zincosomes. Zincosomes are vesicles that can sequester high levels of zinc [17,18,19].

Each of the ZIP and ZnT transporters show tissue specificity and developmental and stimulus responsive expression patterns. On a cellular and subcellular level, they are also localized in specific compartments. Both transporter families respond to various stimuli such as zinc deficiency and excess by displaying specific changes in cellular localization and protein stability [20]. 

There are many zinc transporter mutations that are reported to be involved in inherited diseases. Most notably, a mutation in ZIP4 (SLC39A4), which is an intestinal zinc transporter, is responsible for the rare lethal autosomal-recessive inherited zinc deficiency disease, acrodermatitis enteropathica (AE). AE is characterized by severe dermatological manifestations, gastrointestinal disturbances, weight loss, growth retardation, male hypogonadism, and high susceptibility to infections among other clinical features. Complete recovery occurred after high-dose zinc supplementation [21]. In addition to AE, there is another genetic disease associated to mutations in ZIP transporters. Mutations in SLC39A13, encoding ZIP13, cause a novel subtype of Ehlers-Danlos Syndrome (EDS). EDS is a spectrum of connective tissue disorders caused by mutations affecting collagen synthesis and modification [22].

There are also some genetic diseases affecting the ZnT family. One example is a mutation influencing ZnT2. A substitution of a histidine with an arginine at amino acid 54 (H54R) in the human SLC30A2 gene causes a defect in zinc secretion. The H54R mutation of ZnT2 is autosomal dominant as women who are heterozygous for the allele present the phenotype of reduced zinc production in the breast milk during lactation. Infants that are exclusively breast-fed suffer from zinc deficiency due to low zinc levels in the breast milk, which in turn predisposes them to multiple infections. Zinc deficiency symptoms can be alleviated by oral zinc supplementation to the nursing babies [23].

Expression modulation of ZIPs and ZnTs during inflammatory processes has been documented. Intracellular zinc requirements are altered during an inflammatory event or when an invading pathogen gains access to the cell. For instance, in an allergic inflammation mouse model, expression of zinc transporters was altered, including increases in ZIP1 and ZIP14 and decreases in ZIP4 and ZnT4 [24]. Furthermore, zinc deprivation of phagocytosed *Histoplasma capsulatum* by sequestration into the Golgi apparatus is controlled by ZnT4 and ZnT7 and uptake of extracellular zinc is controlled by ZIP2 [25]. The role of zinc transporters during inflammation and infection is under active investigation and more will be revealed of how those transporters influence zinc homeostasis in different conditions.

### 3.2. Metallothioneins and Other Zinc Binding Proteins

Metallothioneins (MTs) are cysteine-rich 6–7 kDa proteins that bind metal ions such as zinc [26]. Up to 20% of intracellular zinc is bound to MTs, and can be rapidly released. They can form a complex with up to 7 zinc ions. There are four different MT classes; MT-1 and MT-2 are ubiquitous throughout the body, their main function is to maintain cellular zinc homeostasis and chelate heavy metals to reduce cytotoxicity and lower their intracellular concentrations, and due to their reactive oxygen species (ROS) scavenging properties, they help protect against several types of environmental stress. MT-3 and MT-4 expression is restricted to a cell type-specific pattern, with MT-3 predominantly found in the brain and MT-4 is primarily located in stratified epithelial tissues [20,27]. 

In addition to MTs, there are other zinc binding proteins that act as a storage system and control the release of zinc. Albumin binds around 80% of all plasma zinc and is thought to act as a major zinc transporter. Albumin modulates zinc uptake into certain cell types, such as endothelial cells. These cells co-transport albumin-bound zinc via a specific receptor-mediated pathway. Albumin has several metal-binding sites that are specific for different metal ions, for instance, site A binds specifically to zinc at high affinity. However, the fatty acid content of albumin influences metal binding, particularly during conditions of increased free fatty acid mobilization such as after exercise. Zinc-binding capacity may be reduced in such conditions when about four fatty acids may be bound to albumin. Even though zinc albumin complexes do not dissociate as easily as other zinc–protein complexes, they are still considered to have rapid exchange kinetics and contribute to the modulation of free zinc in the plasma [28].

Furthermore, the S100 protein family, which consists of more than 20 members, is composed of EF-hand calcium regulated proteins, and they are distributed in a cell-specific, tissue-specific, and cell cycle-specific manner in humans and other vertebrates. S100 proteins have diverse functions ranging from calcium buffering, intracellular functions such as modulating enzyme activities and secretions, nuclear functions such as apoptosis and transcription, and extracellular activities related to secretion and chemotaxis, among other functions [29]. S100 proteins are also regulated by zinc and several members show higher affinity towards zinc compared to calcium such as the case with S100A3. The zinc-binding site in S100B, S100A6, S100A7, S100A8/A9, S100A12, and S100A15 consist of either three histidine residues and one aspartate or four histidine residues and is distinct from the calcium binding sites in the EF-hands, while S100A2, S100A3, and S100A4 have cysteine-containing zinc-binding sites [30]. A brief description of the role of some S100 protein in nutritional immunity will be mentioned later.

α2-macroglobulin (A2M) is another zinc-binding protein which is an inhibitor of matrix metalloproteases (MMPs) and it is required to remove proteolytic potential, when MMPs increase, forming A2M-proteinase complexes. It has a very high affinity to zinc, where zinc is required for the activation of A2M and also for the binding of A2M with cytokines [31].

The cytokine interleukin 6 (IL6) induces the expression of MT and A2M and consequently reduces zinc availability. IL-6 is released during the acute phase of an inflammatory response. This mechanism is beneficial to the acute immune response, however, a long-term decrease in zinc availability may contribute to pathological processes in conditions of chronic inflammation (e.g., diabetes and dementia). IL6, MT, and A2M expression increase in old age and impaired zinc availability contributes to immunosenescence. A mutation in the IL6 promoter up-regulates its expression leading to increased MT, low plasma zinc, impaired innate immunity and increased risk of Alzheimer disease [32].

## 4. Zinc and Immunity 

Many organs are affected by zinc deficiency, especially the immune system which is markedly susceptible to changes of zinc levels. It seems that every immunological event is influenced by zinc somehow. The immune response involves two mechanisms; innate and adaptive immunity. The first cells to encounter invading pathogens and eliminate them are the cells of the innate immunity. Polymorphonuclear cells (PMNs), macrophages (Mφ), and natural killer cells (NK) are some of those first responders. PMN chemotaxis and phagocytosis are reduced during zinc deficiency, whereas zinc supplementation has the opposite effect. After phagocytosis, pathogens are destroyed by the activity of nicotinamide adenine dinucleotide phosphate (NADPH) oxidases which has been shown to be inhibited by both zinc deficiency and excess [33,34]. Alternatively, PMN can kill pathogens by releasing neutrophil extracellular traps (NETs). These matrices of DNA, chromatin, and granule proteins can capture extracellular bacteria [35]. Chelating free zinc abolishes NET formation was observed in vitro. Moreover, before macrophages can mature into tissue resident cells, circulating monocytes must be attracted to the target tissues and adhere to endothelial cells. In vitro, this process of adhesion is augmented by zinc. Zinc deficiency increases the production of proinflammatory cytokines, such as interleukins IL-1β, IL-6, and tumor necrosis factor (TNF)-α. Recognition of major histocompatibility complex (MHC) class I by NK cells and the lytic activity of NK cells is influenced by zinc depletion. In terms of the adaptive immune response, zinc deficiency causes thymic atrophy and subsequent T-cell lymphopenia as well as reduction of premature and immature B cells, and consequently antibody production is also reduced [36]. The following sections will delve deeper into how zinc influences immune cells and their mediators during inflammation and infection.

It is worth mentioning that most zinc deficiency studies have been conducted on animal models and various cell culture types. Since zinc does not have major storage depot in the body, zinc deficiency is easily and rapidly produced. As mentioned previously, severe zinc deficiency is rare but mild to moderate deficiency is more prevalent. It remains a challenging task to attribute certain clinical manifestations to mild to moderate zinc deficiency, particularly because depletion of zinc from tissue is usually accompanied by deficiencies in other nutrients. This can be further rationalized by understanding the influence of zinc deficiency on food intake; this effect was observed in rats that were either starved or fed zinc deficient diets resulting in reduced weight and metabolic activity among other indicators. Thus common zinc deficiency symptoms can be attributed to either low zinc or to reduced overall nutrient intake [37]. However, that does not undermine the vital role zinc plays in the immune system. 

## 5. Zinc in Inflammation

Inflammation is a natural process required to protect the host from tissue damage and infections, which leads to the resolution of the inflammatory response and the restoration of homeostasis. However, sometimes the inflammation does not resolve and later becomes chronic, leading to loss of tissue function [38]. This section will review how the body mounts an inflammatory response and how zinc influences those processes.

### 5.1. NF-κB and Other Signalling Pathways

Proper modulation of inflammatory pathways is required to achieve adequate response to various stimuli such as stress, free radicals, cytokines, or bacterial and viral antigens. One of the main inflammatory pathways is the nuclear factor kappa-light-chain-enhancer of activated B cells (NF-κB) signaling pathway. It regulates the genes controlling apoptosis, cell adhesion, proliferation, tissue remodeling, the innate and adaptive immune responses, inflammatory processes, and cellular-stress responses. Subsequently, it influences the expression of proinflammatory cytokines such as TNF-α, IL-1β, IL-6, IL-8, and MCP (monocyte chemoattractant protein)-1. NF-κB is one of the most versatile regulators of gene expression [39]. 

The NF-κB protein family in mammalian cells consists of five members, RelA (p65), RelB, c-Rel, p50/p105 (NF-κB1), and p52/p100 (NF-κB2). Different NF-κB complexes are also formed from homo- and heterodimers. Non-active NF-κB complexes are typically found in the cytoplasm, where they are bound to and silenced by a family of inhibitory proteins known as inhibitors of NF-κB (IκBs). There are several IκBs; IκBα, IκBβ, IκBγ, IκBε, Bcl-3 along with p100 and p105 which function as IκB-like proteins that inhibit their NF-κB-subunit dimeric partners, p50 and p52, respectively. Activation of NF-κB requires the phosphorylation of IκBs by the IκB kinase (IKK) complex, which degrades the IκB and releases NF-κB and allows it to translocate freely to the nucleus so it can induce targeted gene expression [39,40]. 

**Figure 1:**
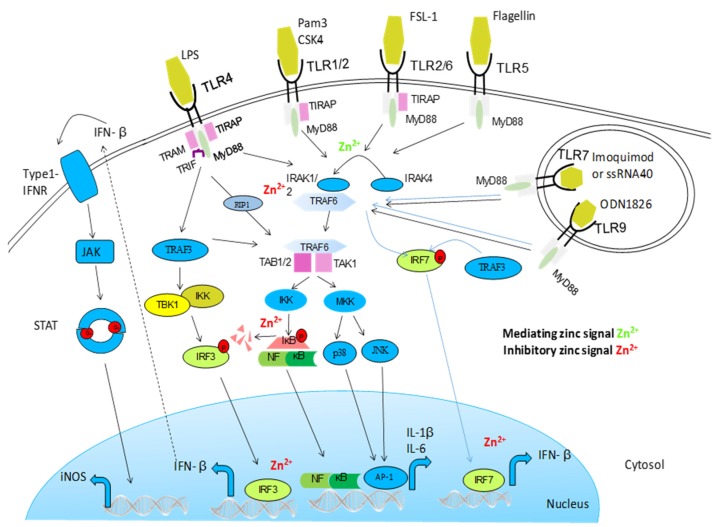
Activation of Toll-like receptor (TLR) signaling pathways is mediated by a complex array of proteins. When TLRs bind ligands, dimerization of the ectodomain of TLRs is induced, bringing their cytoplasmic Toll/IL-1R domains together, resulting in the recruitment of intracellular adapter proteins and initiation of downstream signaling events. Following this, the Toll–IL-1-resistence (TIR) domains of TLRs engage TIR domain-containing adaptor proteins (either myeloid differentiation primary-response protein 88 (MYD88) or TIR domain-containing adaptor protein inducing IFNβ (TRIF) and TRIF-related adaptor molecule (TRAM)). This in turn stimulates downstream signaling pathways that involve interactions between IL-1R-associated kinases (IRAKs) and the adaptor molecules TNF receptor-associated factors (TRAFs), and that lead to the activation of the mitogen-activated protein kinases (MAPKs) JUN *N*-terminal kinase (JNK) and p38, and to the activation of transcription factors. Two groups of transcription factors are activated downstream of TLR signaling; nuclear factor-κB (NF-κB) and interferon-regulatory factors (IRFs). This TLR signaling pathway leads to the induction of proinflammatory cytokines. Zinc influences this pathway on multiple levels. It augments MyD88-dependent signaling whereas inhibiting TRIF-mediated activation of IRF3/7. It has been shown that zinc inhibits IRAK thereby inhibiting further signal transductions. In vitro, zinc also directly impaired LPS-induced IKK activity [45].

There are several conflicting studies regarding the effect of zinc on this process. In vitro studies using different cell types and zinc concentrations as well as the impact of chelating agents have made it obvious that the influence of zinc cannot be interpreted using a unilateral approach [41]. For instance, a study by Haase et al. has revealed that zinc is necessary for the activation of lipopolysaccharide (LPS)-induced NF-κB signaling pathway, whereas chelating zinc with membrane permeable zinc specific chelator TPEN (*N,N,N′,N′*-tetrakis-(2-pyridyl-methyl) ethylenediamine) completely blocked this pathway [42]. On the other hand, there is a growing body of literature that supports the role of zinc as a negative regulator of NF-κB signaling pathways. There are several inhibitory mechanisms that have been suggested. One of the major inhibitory mechanisms relies on how zinc affects the expression of protein A20. A20 is a zinc-finger protein that is recognized as an anti-inflammatory protein which also negatively regulates tumor necrosis factor receptor (TNFR)- and toll like receptor (TLR)-initiated NF-κB pathways (Figure 1). During TNFR signaling, A20 is able to deubiquitinate receptor interacting protein 1 (RIP1), which prevents its interaction with NF-κB essential modulator IKKγ. It also inhibits TLR signaling by removing polyubiquitin chains from TNF receptor associated factor 6 (TRAF6). Although the deubiquitinase activity of A20 remains unchanged by zinc chelator [19], Prasad et al. demonstrated that the induction of A20 mRNA and generation of the protein in pre-monocytic, endothelial, and cancer cell is zinc-dependent [43]. Additionally, zinc supplementation was able to downregulate inflammatory cytokines by decreasing gene expression of IL-1β and TNF-α through upregulation of mRNA and DNA-specific binding for A20, subsequently inhibiting NF-κB activation [44]. 

**Figure 2:**
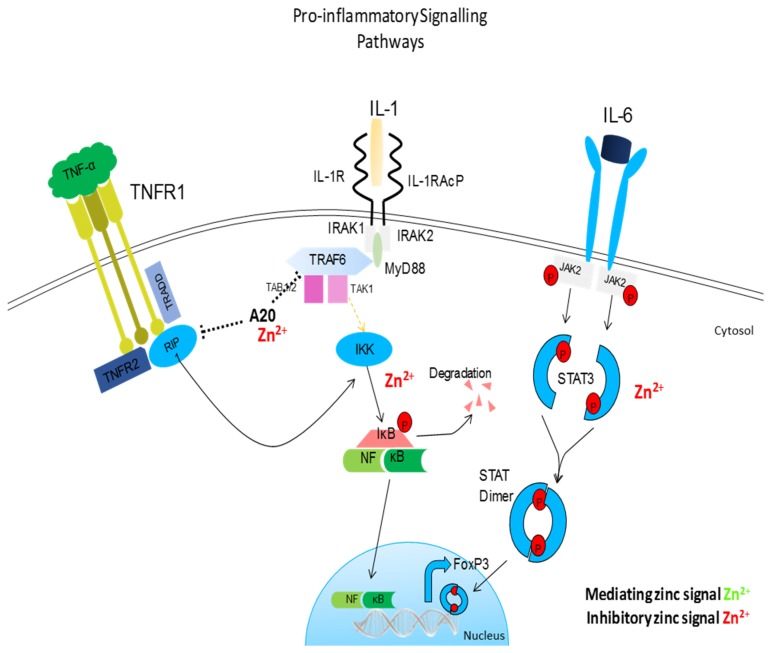
Pro-inflammatory signaling pathway influences by zinc. Similar to TLR signaling, IL-1, and TNF-R signaling pathways converge on a common IκB kinase complex that phosphorylates the NF-κB inhibitory protein, resulting in the release of NF-κB and its translocation to the nucleus. Zinc prevents the dissociation of NF-κB from its corresponding inhibitory protein, thus preventing the nuclear translocation of NF-κB and inhibiting subsequent inflammation. Zinc also inhibits IL-6-mediated activation of STAT3. Zinc acts as anti-inflammatory element influencing major pro-inflammatory signaling pathways.

**Figure 3:**
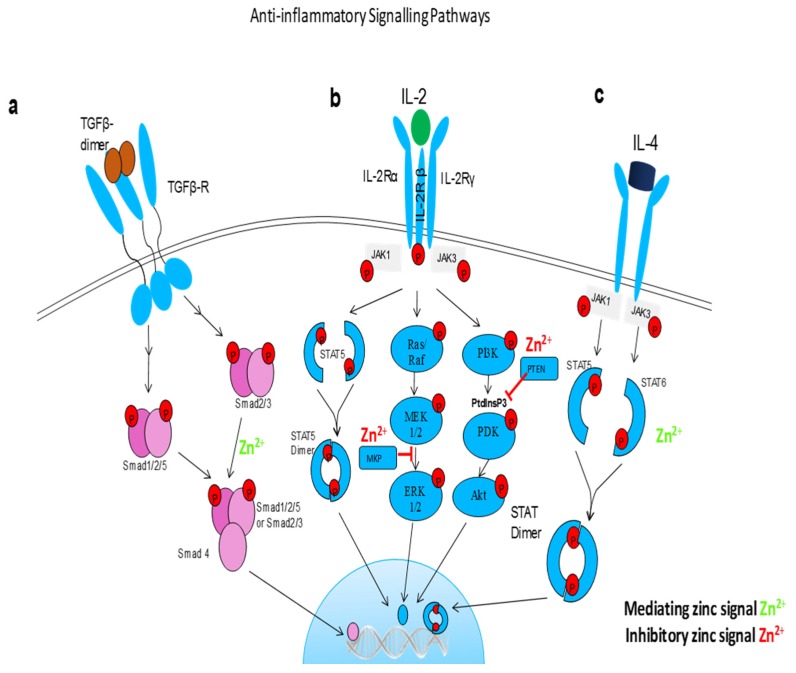
Anti-inflammatory signaling pathways influenced by free zinc. (**a**) TGFβ signaling is dependent on a dynamic on and off switch in Smad activity. Free zinc is a cofactor in Smad proteins and promote Smad 2/3 nuclear translocation and transcriptional activity. (**b**) zinc regulates IL-2 signaling pathway via blocking MAP kinase phosphatase (MKP) in extracellular signal-regulated kinases (ERK) 1/2 pathways and Phosphatase and tensin homologue (PTEN) which opposes phosphoinositide 3-kinase (PI3K) function in PI3k/Akt pathway. (**c**) free zinc phosphorylates STAT6 and promotes translocation of STAT dimers into the nucleus, hence promote the anti-inflammatory effects of Il-4.

There are several studies that have examined the influence of zinc on A20-mediated NF-κB inhibition [46,47,48]. Moreover, zinc inhibits NF-κB activation in the DNA nuclear binding levels by increasing the expression of peroxisome proliferator-activated receptor α (PPAR-α), which is a mediator for lipoprotein metabolism, inflammation, and glucose homeostasis. PPAR-α increase leads to the down-regulation of inflammatory cytokines and adhesion molecule [49]. Additionally, zinc acts as an inhibitor of cyclic nucleotide phosphodiesterase (PDE). When PDE is inhibited, cyclic nucleotide cGMP (Cyclic guanosine monophosphate) is elevated leading to the activation of PKA (protein kinase A) and subsequent inhibition of NF-κB [50]. Similarly, zinc can bind to a zinc finger-like motif found on protein kinase C (PKC) and inhibit PMA-mediated PKC translocation to the membrane. When this occurs in mast cells, NF-κB activity is indirectly inhibited [51]. Those mentioned studies demonstrates how zinc influences inflammation via NF-κB signaling pathways through several mechanisms and at many levels. Figure 2 and Figure 3 provide more details on how zinc influences several pro- and anti-inflammatory pathways.

Zinc can simultaneously modulate inflammation through TLR signaling at different levels and pathways [45]. TLRs are single, membrane-spanning, noncatalytic receptors which are expressed on many cells such as macrophages and dendritic cells and can distinguish and be activated by structurally conserved molecules derived from microbes. In monocytes, it has been observed that TLR4 activation initiates zinc-mediated signaling in a MyD88 (Myeloid differentiation primary response gene 88) and TRIF independent manner. Zinc has been shown to influence cellular signal transduction by inhibition of several dephosphorylating enzymes like protein tyrosine phosphatase (PTPs), cyclic nucleotide phosphodiesterases, and dual specificity phosphatases. Therefore, an alternative mechanism was purposed in which zinc acts as a permissive signal. In this mechanism, the level of intracellular free zinc regulates the rate of dephosphorylation, and thereby the signal intensity of phosphorylation-dependent signaling [19]. 

Zinc also plays a role in modulating the apoptotic and inflammatory processes of caspases, which are a family of endoproteases. Apoptosis is an intricate process where intracellular components are dismantled in a controlled fashion whilst avoiding inflammation and damage to the surrounding cells. Dysregulation in apoptosis influences the pathogenesis of many diseases such as AIDS, diabetes mellitus, autoimmune diseases, malignancies, and most prominently, neurodegenerative disorders. Thus, understanding the regulatory factors involved in this process is crucial for developing therapeutic strategies [52]. There are several conflicting results showing how zinc can be anti- or pro-apoptotic depending on the zinc concentration used. Moreover, it has been observed that both zinc deficiency and supplementation induced apoptosis in the same cellular model [53]. There are two anti-apoptotic mechanisms of action exerted by zinc on caspase-dependent processes. Firstly, zinc suppresses some of the signaling pathways that lead to caspase activation and apoptosis by limiting the damage caused by free oxygen radicals and other toxins. Secondly, zinc directly affects apoptotic caspase enzymes. Influence of zinc on limiting free radicals will be discussed in the following section. With regard to the direct effect zinc has on caspases, many studies established the inhibitory role of zinc on apoptotic caspases 3, 6, 7, 8, and 9 [54,55,56]. Caspase-3 is particularly interesting due to its dominant role in the apoptotic pathway. Zinc is a potent inhibitor of caspase-3 with an IC_50_ below 10 nM [57]. Zinc containing compounds such as ziram (a zinc-containing dithiocarbamate) caused degradation of pro-caspase-1 which is a precursor of the inflammatory caspase-1 [58]. Active caspase-1 cleaves pro-IL-1β and pro-IL-18, which facilitates the secretion of these proinflammatory cytokines [59]. This indicated that zinc can also modulate caspase-controlled inflammatory processes.

Recent findings have established that zinc metabolism and its role in immune function are directly linked to zinc transporter function. This is demonstrated by ZIP8 which functions as a critical negative feedback regulator. When inflammation starts, NF-κB directly activates the expression of ZIP8, which then localizes to the plasma membrane, thereby mediating Zn uptake. When zinc enters the cytosol via ZIP8 it goes on to inhibit IKKβ kinase activity, which leads to the attenuation of the pro-inflammatory response [60].

### 5.2. Oxidative Stress

Under non-pathological conditions, cells produce ROS during cellular respiration. However, excessive production of ROS and the decreased rate of its neutralization and removal by antioxidant defense mechanisms lead to an imbalance between oxidants and antioxidants which results in oxidative stress [61]. Accumulation of those free radicals leads to cell and tissue damage. Oxidative stress is responsible for the development of many chronic diseases such as cancer, cardiovascular disease, atherosclerosis, hypertension, ischemia/reperfusion injury, diabetes mellitus, neurodegenerative diseases (Alzheimer’s disease and Parkinson’s disease), rheumatoid arthritis, and ageing [62].

Within the cell, there are several sites and processes responsible for ROS production, these include; mitochondrial electron transport chain, peroxisomal long-chain fatty acid oxidation, and respiratory burst primarily through activation of NADPHs, which are plasma membrane-associated enzymes. Other enzymes generate ROS during enzymatic reaction cycles, such enzymes are cytochrome P450 monooxygenase, nitric oxide synthase (NOS), xanthine oxidase, cyclooxygenase (COX), and lipoxygenase (LOX). Apart from those endogenous sources of free radicals, there are also exogenous or environmental sources of ROS. Air pollutants, tobacco smoke, ionizing and nonionizing radiations, foods and drugs, as well as xenobiotics can all contribute to oxidative stress [63]. Heavy metals such as lead, arsenic, mercury, chromium, and cadmium; organic solvents; and pesticides are common exogenous sources of ROS [64]. 

In order to neutralize and remove excess ROS, cells have endogenous non-enzymatic and enzymatic antioxidant defense mechanisms. Non-enzymatic antioxidants include glutathione (GSH), thioredoxin (Trx), and melatonin. While Antioxidant enzymatic mechanisms involve enzymes, such as superoxide dismutase (SOD), glutathione peroxidase (GPx), glutathione reductase (GR), catalase (CAT), and heme oxygenase (HO). Exogenous antioxidants include vitamin C, vitamin E, carotenoids including vitamin A, polyphenols including flavonoids and minerals such as zinc, copper, manganese, iron, and selenium [63,65].

Zinc has several antioxidant effects. It is a cofactor of the Cu/Zn-SOD enzyme, which catalyzes the dismutation of superoxide radical (O_2_^•^^−^) into the less harmful O_2_ and H_2_O_2_, which is then detoxified by CAT and GPx. It also inhibits NADPH oxidases, causing reduced generation of ROS. Furthermore, zinc induces the production of MTS, which are excellent ROS scavengers due to the high cysteine content [66]. Stabilization of protein sulfhydryls against oxidation by zinc is another mechanism by which zinc hinders the oxidative processes. Zinc binds directly to the thiol group, creates steric hindrance by binding close to the sulfhydryl group in the protein, and binds to other sites leading to conformational changes in the protein. This all results in the reduced activity of sulfhydryl [67]. Moreover, zinc antagonizes redox-active transition metals such as copper and iron that catalyze formation of free radicals, primarily through Fenton reactions. Those transition metals form complexes with cellular components, such as nucleotides, glucose, and citrates for iron and carbohydrates, DNA and enzymes for copper. When the metal is complexed, it becomes trapped and reacts with H_2_O_2_ and forms reactive hydroxyl radical (HO^•^). This leads to lipid peroxidation, DNA and protein damage, and subsequent severe tissue damage. Zinc is able to replace copper and iron and reduce the localized oxidative injury [68]. 

Zinc supplementation of healthy human subjects reduced the oxidative stress-related by-products malondialdehyde (MDA), 4-hydroxyalkenals (HAE), and 8-hydroxydeoxyguanine in the plasma [44]. This effect was also seen with elderly subjects who usually have lower plasma zinc concentration compared to younger people. Supplementation with zinc has reduced oxidative stress markers and lowered inflammatory cytokines and infection incidence [69]. Involvement of oxidative stress in other processes is discussed in more detail elsewhere [70].

### 5.3. Zinc Status and Inflammatory Cytokines

Zinc can influence the production and signaling of numerous inflammatory cytokines in a variety of cell types. Plasma zinc concentrations rapidly decline during acute phase response to different stimuli such as stress, infection, and trauma. Consequently, zinc is shuttled into cellular compartments, where it is utilized for protein synthesis, neutralization of free radicals, and to prevent microbial invasion. This redistribution of zinc during inflammatory events seems to be mediated by cytokines. Several studies have demonstrated how patients with acute illnesses present with hypozincemia along with elevated cytokine production [71,72].

Chronic inflammation is characterized by increased levels of inflammatory cytokine production. Some conditions are associated with chronic inflammation such as obesity, where patients with lower zinc dietary intake present with lower plasma and intracellular zinc concentrations along with upregulated gene expression of IL-1α, IL-1β, and IL-6 compared to patients with higher zinc intake [73]. 

The effect of zinc deficiency and supplementation is being reported regularly in in vitro and in vivo models. Cell specific responses and varying zinc dosage plays an important role in determining the outcome when subjecting cells to zinc depletion or supplementation. Various study models have been used. There are some noteworthy examples, for instance, the study mentioned above involving elderly subjects revealed that the elderly have significantly lower plasma zinc and higher ex vivo generation of inflammatory cytokines. Zinc supplementation partially reversed the levels of many inflammatory cytokines [69]. Yet zinc supplementation is not always beneficial, adding zinc at very high concentrations (>100 μM) increases cytokine production in some cell types, this was seen in human peripheral blood mononuclear cells (PBMCs) harvested from healthy adults [74] and in human promonocytic HL-CZ cells [75]. As mentioned previously, zinc deficiency induces production of IL-1β. This was seen in LPS-stimulated PBMCs from zinc-deficient adults compared to their zinc-sufficient counterparts [76]. This observation was confirmed through studying the epigenetic mechanisms that are implicated during zinc deficiency and it was reported that long term zinc deprivation promoted changes of the chromatin structures of IL-1β and TNFα promoters enabling the expression of both genes [77]. Additionally, phytohemagglutinin (PHA)-induced production of IL-2 was reduced in PBMCs of zinc-deficient patients with head and neck cancer and in zinc-deficient healthy volunteers [78]. IL-2 and IL-2Rα mRNA were also reduced in zinc deficient elderly people [79]. Similarly, inducing deficiency in healthy men via a zinc restricted diet (4.6 mg/day for 10 weeks) led to a reduction of PHA-stimulated IL-2R production [80]. Furthermore, IL-6 production in zinc-deficient elderly subjects was increased [81]. These observations were later confirmed via DNA methylation profile characterization, which showed that zinc deficiency induced a progressive demethylation of the IL6 promoter in THP1 cells that correlated with increased IL6 expression [82]. 

Cytokines are produced by different types of cells, primarily by T lymphocytes and macrophages. The influence of zinc on these cells may explain the observed effect zinc has on cytokine production. Zinc deficiency affects immune cells in several ways. It reduces thymulin activity, which is required for the maturation of T-helper cells (Th), it causes an imbalance between Th1 and Th2 cells which decrease recruitment of T naïve cells and percentage of cytotoxic T cells [83,84]. It also lowers the cell lytic activity of NK cells [85]. During zinc deficiency, there is also reduced production of IFN-γ and IL-2, which are products of Th1 cells, while the production of IL-4, IL-6, and IL-10 (products of Th2 cells) remain unchanged [86]. On the other hand, zinc excess (50–100 μM) inhibits T cell activity which is demonstrated by the suppression of IL-1β-stimulated IFN-γ expression [87]. This shows how both zinc deficiency and increased zinc levels influence T-cell function. Additionally, regulatory T cells (Treg) are induced and stabilized in zinc-supplemented mixed lymphocyte culture (MLC), in lymphocytes from patients allergic to pollen [88] and in experimental autoimmune encephalomyelitis (EAE) [89]. Furthermore, zinc enhances the capacity of TGFβ1 to induce Treg cells [90,91]. However, the capacity of zinc to induce Tregs is dependent on the activation status of the cells. Activated zinc-supplemented T cells are driven to differentiate to Treg, whereas resting T cells are pushed to Th1 response [89]. Hence, the influence of zinc is expansive yet specific to each cell type.

## 6. Zinc in Infection

### 6.1. Nutritional Immunity

Nutritional immunity is a process by which the host organism sequesters trace minerals during an infection so it can be of limited availability to pathogens. It was first described for iron but has now been extended to other trace elements. Zinc is an essential trace element for both host and pathogens. Pathogens require zinc for survival, propagation and disease establishment. This prompts a competitive process between the host and the invading pathogens. There are three mechanisms by which the host is able to compete for zinc and achieve a zinc limited environment for the pathogen, however, some pathogens have developed tactics to overcome some of those mechanisms [5,92].

On a systematic level, zinc distribution in the body is altered. This mainly involves the small pool of free zinc that is unbound within plasma as 99.9% of zinc remains inside cells and cannot be directly accessed by pathogens. During an infection, plasma zinc levels are markedly reduced. This is achieved by several approaches, but primarily involves the secretion of inflammatory cytokines such as IL-6 that upregulates expression of ZIP14 in hepatocytes which leads to the accumulation of zinc bound to metallothionein in the liver [93]. Furthermore, zinc concentrations can be altered on an extracellular level through the release of some antimicrobial peptides from the S100 family. Several cell types secrete different peptides, keratinocytes secrete S100A7 that can kill *Escherichia coli* by sequestering zinc [94]. Additionally, neutrophils also secrete calprotectin (a heterodimer of S100A8 and S100A9). It can inhibit the growth of *Staphylococcus aureus* by sequestering zinc as well [95]. As mentioned previously, neutrophils also release NETs, in which they cast out DNA, chromatins, and granular proteins to capture and kill microorganisms [35]. Calprotectin is also released in very high concentrations during NETosis, where it is either incorporated into NET or in the surrounding fluids. On an intracellular level, macrophages have evolved two opposing strategies to kill phagocytosed pathogens. Macrophages can deprive *Histoplasma capsulatum* of zinc by reducing the phagosome zinc content [25]. On the other hand, they kill *Mycobacterium tuberculosis* by intoxicating it with excess amounts of zinc and copper [96].

Some pathogens have developed defense strategies against several of those mechanisms. For example, *Neisseria meningitidis* uses a high affinity zinc uptake receptor ZnuD which allows it to escape NET-mediated nutritional immunity [97]. It also responds to zinc deprivation by expressing CbpA, which is an outer membrane protein that acts as a receptor for calprotectin and enables *N. meningitides* to acquire zinc bound to calprotectin. *N. meningitidis* effectively undermines an important defense mechanism used by the host and utilizes it in its favor [98]. *Yersinia pestis* utilizes the siderophore yersiniabactin (Ybt) as a zincophore, and ZnuABC, which is the high-affinity zinc transporter found in bacteria and fungi, to acquire zinc and develop a lethal infection in a septicemic plague mouse model [99]. *Salmonella typhimurium* can also overcome calprotectin-mediated zinc chelation by expressing ZnuABC, thereby allowing it to compete with commensal bacteria and thrive in the inflamed gut [100].

Although low zinc is associated with an impaired immune system and poor prognosis in conditions such as sepsis, supplementation during sepsis was not beneficial, whereas prophylactic zinc administration showed some positive results [101]. In contrast, administering zinc via lozenges at concentrations of ≥75 mg/day reduced the duration of common cold symptoms in healthy individuals [102]. It is worth mentioning that the decision to administer zinc during an infection must take into account the risks of creating a zinc microenvironment that is more favorable for pathogen growth, meanwhile downgrading the efforts carried out by the innate system to reduce free zinc. This only demonstrates the needs for more research to investigate and understand the therapeutic potentials of zinc for infectious diseases.

Despite zinc being recognized as a crucial factor for the proper functioning of the immune system, increased susceptibility to infections and other immune dysfunctions could be the consequence of one or more nutrient deficiencies. The same dietary factors leading to deficiency of one micronutrient often cause deficiency of other micronutrients. Additionally, supplementation of one micronutrient in high doses may exacerbate the status of other micronutrients, which can be seen in the case of iron supplementation impairing zinc uptake. Furthermore, excess supplementation of either zinc or copper influences the tightly controlled ratio of those two metals. Supplementation with high doses of zinc for prolonged periods lead to copper deficiency [53]. Also, altered zinc and copper homeostasis is implicated in several conditions, for example, high copper/zinc ratio was observed in the elderly, particularly those with neurodegenerative disorders [103,104]. The copper/zinc ratio maybe considered a biomarker for mortality in the elderly population [105], which further emphasizes the importance of understanding the complex interplay between different nutrients and their collective influence on health. Maintaining the homeostasis of micronutrients and vitamins is fundamental for a healthy immune response and overall wellness of the body. However, the interaction between micronutrients must be explored further in order to design supplementation interventions that target multiple deficiencies.

Table 1, Table 2 and Table 3 provide an accumulative record of selected publications on zinc supplementation trails. The influence of zinc on viral (Table 1), bacterial (Table 2), and parasitic (Table 3) infections depends on many factors that include, but not limited to; zinc status at baseline measurement, zinc supplementation concentration and frequency, zinc species and age. Results must be interpreted cautiously and analytically. A continuous joint effort between members of the scientific community who conduct such clinical trials will only shed more light on the influence zinc has on infectious diseases and how it can be utilized further.

### 6.2. Zinc as a Critical Component of the Membrane Barrier

Zinc may contribute to the host defense by maintaining the membrane barrier structure and function. This is very crucial in sites like the lung and intestine that become continuously exposed to a myriad of pathogens and noxious agents. Zinc deficiency may contribute to the severity of infectious diseases and mortality in malnourished children. In case of diarrhea, which is the leading cause of death globally among children under five years of age, zinc supplementation has been shown to reduce the severity and duration of symptoms [146]. WHO recommends zinc supplementation of 20 mg per day for 10–14 days for the management of diarrhea [147]. Several studies have examined the influence of zinc depletion and supplementation on the permeability of the endothelial cell barrier. The intestinal epithelium barrier consists of intercellular junctional complexes between neighboring cells that provide a continuous seal around the apical region of the cells. These complexes are composed of several units, including the tight junctions (TJ) and adherens junctions (AJ), that form circumferential zones of contact between adjacent cells. E-cadherin is the main transmembrane adhesion molecule localized at the AJ and its binding to β-catenin is fundamental for appropriate AJ organization. Zinc depletion disrupted the TJ and AJ through several mechanisms. One way zinc affected structural proteins was by enhancing the degradation of E-cadherin and β-catenin [148]. This was also seen in zinc-deprived airway epithelial cells, where there was accelerated proteolysis of E-cadherin and β-catenin leading to increased leakage across the monolayer of upper and alveolar lung epithelial cultures [149]. Hypozincemia may induce uncontrolled neutrophil migration through the disrupted junctional complexes by inducing chemokine production. Exacerbated inflammation may develop and lead to mucosal damage which further contributes to intestinal and lung disease. On the other hand, zinc supplementation has been seen to preserve and restore membrane function and structure [148]. 

Zinc is also an integral part of the epidermal and dermal tissues, where it acts as a stabilizer of cell membranes and as an essential cofactor in numerous transcription factors and enzymes. This includes the zinc-dependent matrix metalloproteinases that enhance autodebridement and keratinocyte migration during wound repair. Moreover, zinc confers resistance to epithelial apoptosis through cytoprotection against reactive oxygen species and bacterial toxins possibly through antioxidant activity of the cysteine-rich metallothioneins [150]. Hence, zinc deficiency may result in delayed wound healing, particularly in the elderly with impaired nutritional status. Delayed wound healing in the elderly constitutes a major clinical and economic challenge, especially as the aging population grows [151]. Topical zinc therapy has shown promising results in enhancing wound healing due to zinc’s role in reducing superinfections and necrotic material by augmenting local defense systems and collagenolytic activity, as well as promoting epithelialization of wounds [150]. Thus, utilizing topical zinc treatments to support wound healing provides a therapeutic advantage and enhances quality of life.

### 6.3. Peptidoglycan Regulation Proteins (PGLYRPs)

Another beneficial effect that zinc has on secretory molecules is how it is involved in the bactericidal activity of peptidoglycan recognition proteins (PGRPs or PGLYRPs). Those innate immunity pattern recognition molecules have effector functions and are expressed in either PMN molecules or in the skin, eyes, salivary glands, throat, tongue, esophagus, stomach, and intestine. Along with other antimicrobial peptides they protect the body from pathogen at the first line of exposure [152]. The activity of PGLYRPs against Gram-positive and Gram-negative bacteria is dependent on free zinc [153]. 

## 7. Conclusions

Zinc is a multipurpose metal that is vital for the growth and function of all cells. The immune system is especially affected by the modification of zinc homeostasis. Achieving an optimal immune response to different stimuli and avoiding damage of tissues and organs is a delicate balance that relies, amongst other factors, on the regulation of zinc in extracellular and intracellular compartments. 

It would be highly beneficial to attain a standard by which zinc supplementation can be administered in different conditions, however, creating such standard might be challenging due to many conflicting findings on various cell types, experimental models, and zinc concentrations, and due to the lack of a good biomarker for zinc status in the body. Nevertheless, resolving zinc deficiency and the adverse manifestations associated with it can be achieved through adequate diet and supplementation. 

## Figures and Tables

**Table 1:** Zinc supplementation and viral diseases (updated from [106]).

Disease	Zinc Species	Zinc Dosage	Period	Participation	Effect of Zinc Supplementation	Reference
**Common cold**	more than 12 different studies, analyzing the therapeutic effects of zinc				variable results, reduced duration of symptoms if administered within 24 h of onset	[107]
	zinc sulfate	15 mg daily	7 months	100 (Z), 100 (P)	lower mean number of colds demonstrating the prophylactic effect of zinc	[108]
**HIV/AIDS**	Not specified	12 mg for women and 15 mg for men/day	18 months	115 (Z) 116 (P)	no effect on viral load. four-fold reduction in the likelihood of immunological failure. Reduced the rate of diarrhea by more than half. No significant difference in mortality	[109]
	Chelated zinc	15 mg daily	12 months	Low: 5 (Z)/7 (P) Normal: 8 (Z)/10 (P)	CD4^+^ cell count significantly increased	[110]
	Zinc sulfate	20 mg daily	24 weeks	26 (Z) 26 (P)	no effect on the increase in CD4%, decrease in viral load, anthropometric indices, and morbidity profile in HIV-infected children started on ART	[111]
	Zinc sulfate	45.5 mg daily	1 month	29 (Z) 28 (P)	increase or stabilization in body weight; increase in plasma zinc levels, CD4^+^ T cells and plasma active zinc-bound thymulin; reduced or delayed frequency of opportunistic infections due to *Pneumocystis jiroveci* and *Candida*, not to *Cytomegalovirus* and *Toxoplasma*	[112]
	Zinc gluconate	45 mg three time daily	15 days	5 (Z) 5 (C)	increased zinc concentrations in red blood, HLA-DR + cells, stimulation of lymphocyte transformation and phagocytosis of opsonized zymosan by neutrophils	[113]
	Zinc sulfate	10 mg daily	6 months	44 (Z) 41 (P)	no effect on HIV viral load; decreased morbidity from diarrhea	[114]
	Zinc sulfate	50 mg daily	1 month	31 (Z) 34 (P)	no improvements in immune responses to tuberculosis, CD4/CD8 ratio, lymphocyte subsets, and viral load	[115]
	Zinc sulfate	25 mg daily	6 months	200 (Z) 200 (P)	when supplemented to pregnant HIV-positive women, no effect on birth outcomes or T-lymphocyte counts, and negative effects on hematological indicators	[116]
	Zinc sulfate	25 mg daily	6 months	200 (Z) 200 (P)	increased risk of wasting	[117]
				50 (Z) 50 (P)	no effect on viral load	
	Zinc gluconate	50 mg daily	6 days	44 (Z) 45 (P)	no improvements in antibody responses to a pneumococcal conjugate vaccine	[118]
**hepatitis C virus**	Not specified	10 mg	60 days	26 (Z + 6400 mg/day Branched-chain amino acids) 27 (P)	BCAA-to-tyrosine ratio (BTR) and zinc levels were significantly increased compared with the placebo group. supplementation reduced the serum α-fetoprotein AFP levels in patients who had elevated serum AFP levels at baseline	[119]
	Polapre-zinc	150 mg	48 weeks	11 (Z) 12 (C)	serum alanine aminotransferase (ALT) level is lower in zinc group compared to control group. HCV RNA disappeared in all patients in the zinc group and in 80% control patients at 48 week. Polaprezinc supplementation decreased plasma thiobarbituric acid reactive substances and prevented the decrease of polyunsaturated fatty acids of erythrocyte membrane phospholipids	[120]
	Polapre-zinc	17 mg twice a day	24 weeks	40 (Z) 35 (C)	zinc supplementation increases serum zinc levels and improves the response to IFN-α therapy	[121]
	Zinc gluconate	50 mg daily	6 months	18 (Z) 35 (P) 20 (C)	increased serum zinc levels; decreased incidences of gastrointestinal disturbances, body weight loss, and mild anemia	[122]

Z—zinc, P—placebo, C—control.

**Table 2:** Zinc supplementation and bacterial infectious diseases (updated from [106]).

Disease	Zinc Species	Zinc Dosage	Period	Participation	Effect of Zinc Supplementation	Reference
**Diarrhea**	multiple different studies				decreased duration, severity and occurrence of diarrhea	[123]
	Not specified	20 mg daily	14 days	41 (Z) 39 (micronutrient combination * + Vit A) 44 (Z+ Vit A) 43(P)	supplementation with a combination of micronutrients and vitamins was not superior to zinc alone, confirming clinical benefit of zinc in children with diarrhea	[124]
**Respiratory tract infections**	Zinc sulfate	20 mg daily	5 months	134 (Z) 124 (P)	reduced acute lower respiratory tract infection morbidity	[125]
	zinc gluconate	10 mg daily	60 days	48 (Z) 48 (P)	reduced episodes of acute lower respiratory infections and severe acute lower respiratory infections. Increased infection free days	[126]
	Zinc oxide	5 mg daily	12 months	162 (Z) 167 (C)	decreased incidence of upper respiratory tract infections and diarrhoeal disease episodes	[127]
	zinc gluconate	10 mg daily	6 months	298 (Z) 311 (P)	increased plasma zinc levels; decreased episodes of infection	[128]
	Zinc acetate	10 mg twice a day	5 days	76 (Z) 74 (P)	increased serum zinc levels and recovery rates from illness and fever in boys	[129]
	Zinc sulfate	15 mg daily	6 months	40 (Z) 40 (P)	increased plasma retinol concentrations; earlier sputum conversion and resolution of X-ray lesion area	[130]
**Tuberculosis**	zinc sulfate	220 mg daily	18 months	8 (Z)	reduced dose of clofazimine; withdrawal of steroids; toleration of dapsone; reduced incidence and severity of erythema nodosum leprosum; gradual decrease in the size of granuloma; gradual increase in the number of lymphocytes	[131]
**Lepromatous leprosy**	zinc sulfate	220 mg daily	18 months	15 (Z) 10 (P)	decreased erythema, edema, and infiltration; regrowth of eyebrows; reduced bacterial index of granuloma; increased serum zinc levels, neovascularization, and endothelial cell proliferation	[132]
	Zinc acetate	200 mg twice a day	13 weeks	17 (Z) 10 (P) 10 (C)	increased serum zinc levels and delayed hypersensitivity reactions; decreased size of skin nodules; disappearance of erythema; regrowth of eyebrows	[133]
	zinc sulfate	220 mg daily	4 months	40 (Z)	improvements on frequency, duration, and severity of erytheme nodosum leprosum reactions; reduction in steroid requirement	[134]
**Shigellosis**	zinc acetate	1.3 mg/kg three times a day	1 month	16 (Z) 16 (P)	increased intestinal mucosal permeability and better nitrogen absorption; increased serum zinc and alkaline phosphatase activity	[135]
	zinc acetate	20 mg daily	2 weeks	28 (Z) 28 (P)	increased serum zinc levels, lymphocyte proliferation in response to phytohemagglutinin and plasma invasion plasmid-encoded antigen-specific IgG titers	[136]
	zinc acetate	20 mg daily	2 weeks	28 (Z) 28 (P)	increased serum zinc levels, serum shigellacidal antibody titers, CD20^+^ cells, and CD20^+^CD38^+^ cells	[137]
	Not specified	20 mg daily	2 weeks	14 (Z) 16 (C)	faster recovery from acute illness. Increased mean body weight. Fewer episodes of diarrhoea	[138]
**Helicobacter pylori infection**	polapre zinc	150 mg twice a day	7 days	33 (Z) 28 (C)	administration of zinc together with antimicrobial therapy increased cure rate of *Helicobacter pylori* infection compared with antibiotic treatment alone	[139]

Z–zinc, P–placebo, C–control; Diarrhea and respiratory infections can be caused by nonbacterial pathogens. The mentioned studies do not specific the causative agent; * micronutrient combination: zinc, 20 mg; iron, 10 mg; copper, 2 mg; selenium, 40 mg; vitamin B12, 1.4 mg; folate, 100 mg.

**Table 3:** Zinc supplementation and parasites (updated from [103]).

Disease	Zinc Species	Zinc Dosage	Period	Participation	Effect of Zinc Supplementation	Reference
**Malaria**	Not specified	10 mg 6 times/week	6 months	74 (Z + 1 single dose of 200 000 IU Vit A) 74 (P)	significant decrease in the prevalence malaria. Lower malaria episodes. Time to first malaria episode was longer. 22% fewer fever episodes than the placebo group	[140]
	Zinc gluconate	10 mg 6 times/week	46 weeks	136 (Z) 138 (P)	reduction in *Plasmodium falciparum*-mediated febrile episodes	[141]
	Zinc acetate/zinc gluconate	70 mg twice a week	15 months	55 (Z) 54 (P)	not statistically significant trend towards fewer malaria episodes; no effect on plasma and hair zinc, diarrhea, and respiratory illness	[142]
	Zinc sulfate	12.5 mg 6 times/week	6 months	336 (Z) 344 (P)	increased serum zinc levels; reduced prevalence of diarrhea	[143]
	Zinc sulfate	20 or 40 mg daily	4 days	473 (Z) 483 (P)	increased plasma zinc, no effect on fever, parasitemia, or hemoglobin concentration	[144]
	Zinc sulfate	20 mg daily	7 months	191 (Z) 189 (P)	no significant effect on P. vivax incidence; significantly reduced diarrhea morbidity	[145]

Z–zinc, P–placebo.
